# Review: distributed time-domain sensors based on Brillouin scattering and FWM enhanced SBS for temperature, strain and acoustic wave detection

**DOI:** 10.1186/s43074-021-00038-w

**Published:** 2021-07-30

**Authors:** Xiaoyi Bao, Zichao Zhou, Yuan Wang

**Affiliations:** 1 School of Electrical Engineering and Computer Science, University of Ottawa, Ottawa, Ontario K1N 6N5 Canada; 2 Department of Physics, University of Ottawa, 25 Templeton Street, Ottawa, Ontario K1N 6N5 Canada

## Abstract

Distributed time-domain Brillouin scattering fiber sensors have been widely used to measure the changes of the temperature and strain. The linear dependence of the temperature and strain on the Brillouin frequency shift enabled the distributed temperature and strain sensing based on mapping of the Brillouin gain spectrum. In addition, an acoustic wave can be detected by the four wave mixing (FWM) associated SBS process, in which phase matching condition is satisfied via up-down conversion of SBS process through birefringence matching before and after the conversion process. Brillouin scattering can be considered as the scattering of a pump wave from a moving grating (acoustic phonon) which induces a Doppler frequency shift in the resulting Stokes wave. The frequency shift is dependent on many factors including the velocity of sound in the scattering medium as well as the index of refraction. Such a process can be used to monitor the gain of random fiber laser based on SBS, the distributed acoustic wave reflect the distributed SBS gain for random lasing radiation, as well as the relative intensity noise inside the laser gain medium. In this review paper, the distributed time-domain sensing system based on Brillouin scattering including Brillouin optical time-domain reflectometry (BOTDR), Brillouin optical time-domain analysis (BOTDA), and FWM enhanced SBS for acoustic wave detection are introduced for their working principles and recent progress. The distributed Brillouin sensors based on specialty fibers for simultaneous temperature and strain measurement are summarized. Applications for the Brillouin scattering time-domain sensors are briefly discussed.

## Introduction

Over the past decades, distributed Brillouin scattering based temperature and strain sensing have found many applications in structural health monitoring of bridges, dams, pipelines and railways. Compared to the Rayleigh scattering or Raman scattering based distributed temperature sensors, Brillouin scattering based distributed sensors provide a high spatial resolution over long sensing ranges [1–3] due to the high SBS gain from narrow linewidth (MHz) and low power requirement for the high gain amplification, which enables short interaction length with relative stronger signal comparing with Raman amplification with broadband (THz). The Brillouin scattering is the inelastic process by which light waves are scattered by acoustic phonons. It can be considered as the scattering of a pump wave from a moving grating (acoustic phonon) which induces a Doppler frequency shift in the resulting Stokes wave. The frequency shift is dependent on many factors including the velocity of sound in the scattering medium as well as the index of refraction. The distributed time-domain sensing employs a narrow optical pulse as the probe signal to interrogate the acoustic wave in the optical fiber, and the location is mapped through the flight time of the pulse from the source to the fiber location. There are two typical distributed time-domain configurations that commercially available based on Brillouin scattering: Brillouin optical time-domain analysis (BOTDA) [4, 5] and Brillouin optical time-domain reflectometry (BOTDR) [6, 7]. Compared with BOTDR, BOTDA uses the stimulated Brillouin scattering (SBS) to get a strong signal and better spatial resolution by sending both pulsed probe light and continuous pump light in the fiber with an enhanced scattering process. The performance of BOTDA has been improved significantly over the years which lead to applications in the structural health monitoring in the industry [8–10]. Since the first demonstration of the distributed temperature sensor based on Brillouin scattering [11] with 3 °C temperature resolution, 100 m spatial resolution over 1 km sensing length, many researchers around the world have contributed to this field to demonstrate long sensing length (over 100 km), higher spatial resolution (cm) and high temperature resolution (<1 ° *C*) and strain resolution $\left(\sim 10\mu \varepsilon, 1\mu \varepsilon ={10}^{-6}\frac{\delta l}{L}\right)$ [12]. The significant improvements brought by the distributed fiber sensors community have made the technology to almost maturity through laboratory, physical models, and in-situ tests, which have attracted applications in civil engineering [13] for many field demonstration of structural health monitoring. Geo-hydrological monitoring [14] is also benefited from distributed fiber sensors, where the optical fibers are placed in soil levees, slopes/landslides, and ground subsidence that formed a significant amount of current geohazards, as well as for environmental geophysical and mining engineering. The BOTDA sensors can reach centimeters spatial resolution with several tens of kilometres sensing range, which makes it a perfect tool to monitor the health of tunnel, bridge, oil pipeline, railways and so on. The key challenge is to find a method that is capable to locate stress points or hot spots at any point along with the sensing fiber with high sensitivity and spatial resolution, yet within acceptable speed for dynamic strain, and temperature detection, as well as at cheaper price.

The Brillouin scattering based temperature and strain sensors are achieved by measuring the reflected spectrum of the acoustic wave induced by Brillouin dynamic grating (BDG) [15]. It is generated by two counter-propagating pump waves through the SBS process as a periodically modulated refractive index associated with an acoustic wave via electrostriction effect. BDG is a moving grating with a lifetime of about 10 ns, and it has a Brillouin frequency shift relative to the reflected light of the probe beam. In most BDG experiments, a high-birefringence polarization-maintaining fiber (PMF) is used [16, 17], where two pump waves are launched into one axis to create a Brillouin grating and a probe wave is launched into the other axis to read the grating; when the frequency difference between the probe wave and the pump waves satisfies the phase matching condition, a maximum reflection on the Brillouin grating can be observed at phase matching condition of FWM process: two pump waves, a probe wave and a reflected wave. It is up or down conversion of Brillouin scattering. The BDG is a weak grating, which can be enhanced significantly by the formation of the random fiber laser based on SBS gain in optical fiber [18]. The enhanced BDG via random SBS is formed in one axis, and the probe beam is launched in the other axis, and the distributed reflection of the probe beam and random SBS laser via excited acoustic wave can be detected at phase matching condition related wavelength for the maximum reflection at the highest SBS gain location. The distributed random SBS laser reflection provides (1) the distributed gain which is proportional to the spatially changed random laser intensity; (2) the local birefringence modulation due to random SBS laser; (3) the linewidth of the acoustic wave when self-heterodyne detection is implemented. Instead of distributed sensing of the temperature and strain change induced by BDG, we detected the distributed noise initiation, formation, amplification in random SBS laser via detection of the excited acoustic wave at every locating in SBS gain fiber under mismatching of FWM relative maximum gain condition. This allows time and position dependent intensity fluctuation monitoring in random fiber laser based on SBS gain.

This paper reviews the recent advancement in Brillouin scattering based BOTDA and BOTDR systems for distributed temperature and strain measurement, FWM enhanced SBS in acoustic wave detection in random SBS laser, and simultaneous temperature and strain measurement using specialty fibers. The influence factors to the performance of the Brillouin based sensors have been summarized, so that readers will have a comprehensive understanding of the advantages and limitations of this technology.

The layout of the paper is arranged as follows: the introduction to the distributed Brillouin fiber sensors is presented in Section 1, the theory of the temperature and strain dependence of the Brillouin frequency shift, and theoretical analysis of the SBS and four-wave mixing enhanced SBS are also provided in Section 1.1. Principles of BTODA and BOTDR are presented in Section 2 and Section 3 respectively. Section 4 will introduce the FWM enhanced SBS and its applications in acoustic wave detection in random SBS laser. The general parameters of the Brillouin based sensors and limitations are introduced in Section 5 and Section 6. Simultaneous temperature and strain measurement using specialty fibers based on Brillouin scattering is presented in Section 7. Some applications are presented in section 8 and section 9 is the conclusion.

## Theoretical analysis

### Temperature and strain dependence of the Brillouin frequency shift (BFS)

The time-domain distributed sensing employs the principle known as of time-of-flight. The technology itself is comparable to that utilized by RADAR or in any modern rangefinder. There is no specialized sensing point detection requirement, thus making it a very simple and effective system for measuring over large distances. Consequently, fiber distributed sensors take advantage of the reflection characteristics of laser light travelling down an optical fiber, vary only with factors that affect the optical fiber itself, such as temperature, strain or sound via the sound wave velocity given by [19]: ${V}_a=\sqrt{\frac{Y\left(1-\kappa \right)}{\left(1+\kappa \right)\left(1-2\kappa \right)\rho }}$, where *Y* is the Young’s modulus, *κ* is the Poisson’s ratio, and *ρ* is fiber density. The relation between the Brillouin frequency shift (BFS) *υ*_*B*_ and the acoustic velocity *V*_*a*_ is given by [20] ${\upsilon}_B=\frac{2{V}_a{n}_{eff}}{\lambda_p}$, where *λ*_*p*_ is the wavelength of the pump laser, *n*_*eff*_ is the effective refractive index of the optical fiber. The temperature *T* [11] and strain *ε* [21] dependence of the BFS relative to reference temperature *T*_0_ and a reference strain *ε*_0_ can be written as [12].

$$ {\upsilon}_B\left(T,\varepsilon \right)-{\upsilon}_B\left({T}_0,{\varepsilon}_0\right)={C}_T\left(\mathrm{T}-{T}_0\right)+{C}_{\varepsilon}\left(\varepsilon -{\epsilon}_0\right) $$

The strain value is often expressed as micro-strain: $\mu \varepsilon ={10}^{-6}\varepsilon =\frac{\Delta L}{L},$ where Δ*L* and *L* are the relative length change and original length, respectively. *C*_*T*_ and *C*_*ε*_ are the temperature and strain coefficients, they change with the fiber parameters, such as core, cladding and jacket, as well as wavelength of the pump laser. The Brillouin frequency shift is material dependent and associated with density fluctuations of the medium [12], which can be influenced by environmental perturbations such as temperature variations or mechanical stresses. As a good approximation for SMF28 fiber at 1550 nm around room temperature: ∆*υ*_*B*_~1.2*MHz*/ ° ∁, and the strain change: ∆*υ*_*B*_~20*μϵ*/*MHz*, both values can be used to convert the measured BFS to the change of the local temperature or strain. The Brillouin frequency in optical fiber is in the range of 9-20GHz at the wavelength range of 1200-1600 nm.The BFS can be obtained by measuring Brillouin gain spectrum (BGS) or Brillouin loss spectrum [22], which represent the Brillouin interaction in each of the fiber sections. In the Brillouin gain based configuration, a continuous wave (cw) Stokes wave counter-propagates with a pulsed pump wave. Throughout the sensing fiber, energy is transferred from the pump pulse to the Stokes wave via the Brillouin interaction. There is a significant drawback to this technique in that the short pump pulse has very little energy compared to the cw Stokes, and the pump wave will quickly become depleted due to the energy conversion. In the Brillouin loss configuration [20], the roles of the pump wave and Stokes wave are reversed. The Stokes wave is now pulsed and the pump wave is cw and is the waveform detected at the output. In this configuration, the energy in the pump wave is much larger and loss to the Stokes wave will not result in significant depletion for short fiber lengths [12]. The advantage of this technique is that much longer sensing lengths become possible. In fact, Brillouin loss based sensing systems demonstrate an overall better performance than the Brillouin gain based one for long range sensing and are more favorable for industrial applications, for instance commercial instruments specify a 30 km range with 1.5 m spatial resolution (Omnisens DITEST-STA).

BGS can be obtained by scanning the pump and probe wave frequency difference in a range of a few hundred to GHz around estimated BFS, depending on the range of temperature and strain variations. The local BFS is obtained by fitting the BGS and pick the peak value, such a fitting process can help to remove the system noise induced error in peaking frequency determination [23]. The principle of reconstructing the local BGS in the time-domain can be classified as Brillouin optical time-domain analysis (BOTDA) based on SBS amplification, which requires two ends access to optical fiber, and Brillouin optical time-domain reflectometry (BOTDR) which requires one-end access to optical fibers. Normally, reflectometry of one-end access is easier for applications due to simple setup, however the weak spontaneous Brillouin scattering signal made the distributed senor having low signal to noise ratio which comes at the cost of measurement accuracy, such as low temperature, strain and spatial resolution, especially for long range (> 100 km) which also means long averaging time associated with long measurement time.

### Brillouin scattering in time domain distributed fiber sensors

Brillouin scattering is caused by the collective acoustic oscillations of the solid state matter. The intermolecular interaction in the solid state matter (glass fiber) creates a tendency for molecules to stay at a stable separation distance from each other. There is an energy penalty when the actual intermolecular distance is either farther apart or closer than this stable separation. The existence of balanced intermolecular distances would set a new collective motion. If a neighboring molecule becomes closer than stability allows, and then it will be pushed away towards a new point of stability. However, when it reaches that position it will not stop; rather it will overshoot passing the stable separation distance. Once it is farther away it will experience an attraction to pull it back toward the optimum position. However it will again overshoot when it returns. Such a repeating cycle forms a collective motion called acoustic phonons [24]. Such a physics process can be described by the three wave coupled equation for pump, probe also called Stokes wave, if the frequency difference between pump and Stokes wave is close to the acoustic wave frequency of the medium, which ensures wave coupling among three waves, and acoustic wave [25]:

1a $$ \frac{\partial {E}_p}{\partial z}+\frac{n}{c}\frac{\partial {E}_p}{\partial t}= i\kappa {E}_s\rho $$

1b $$ -\frac{\partial {E}_s}{\partial z}+\frac{n}{c}\frac{\partial {E}_s}{\partial t}= i\kappa {E}_p{\rho}^{\ast } $$

1c $$ \frac{\partial \rho }{\partial t}+\left[\frac{1}{2}{\Gamma}_B+i\left({\Omega}_B-\Omega \right)\rho \right]=i{\gamma}_e{E}_p{E}_s^{\ast } $$

Where *E*_*p*_*, E*_*s*_ *and ρ* are defined as the field amplitudes of the pump wave, probe wave and the acoustic wave, respectively; Ω_*B*_ is the angular frequency of the BFS (Ω_*B*_ = 2π *ν*_*B*_); Ω is the angular frequency offset between the pump and probe waves; Γ_*B*_ is the linewidth of the Brillouin gain spectrum (BGS); κ and *γ*_*e*_ represent the elasto-optic and electrostrictive coupling coefficients, respectively. Electrostriction effect exists in all materials and it consists of a mechanical displacement as a response to an electric field, in here we refer to SBS. Electrostrictive pressure results from the propagation of two lightwaves in a fiber medium. The frequency difference between two optical waves equals the induced acoustic wave frequency, namely, Brillouin frequency. The fiber attenuation is ignored in the above equations for a short fiber length.

Therefore, the accurate and general solutions of the coupled three-wave equations can be obtained by employing the numerical method [26]. The above equations form the foundation for the Brillouin gain and Brillouin loss (when –*κ* is applied in above equations) formulas presented in following sections for Brillouin scattering based distributed sensors.

### Four wave mixing enhanced SBS

Four-wave mixing (FWM) based on SBS was studied to produce very high reflection coefficient, called Brillouin-enhanced four-wave mixing (BEFWM) [27]. In this technique, the signal beam and one of the pump beams have a frequency difference close to Brillouin frequency. This excites acoustic wave that scatters the second pump beam to produce a conjugate beam with high reflectivity. With the high pump intensities, the reflectivity of the conjugated beam is proportional to the ratio of the pump beam intensities inside crystal [28]. This principle has been applied in the fiber to produce Brillion dynamic grating (BDG) in polarization maintaining fiber (PMF) [15]. BDG has been used for temperature and strain measurement, which can discriminate temperature and strain for Brillouin-based sensors [29, 30].

The frequency difference of pump and Stokes waves is near Brillouin frequency, which is counter propagating along one axis of the PMF; a probe beam polarized along the orthogonal axis illuminates the moving grating induced by the pump and Stokes waves; when the phase-matching condition is satisfied, a diffracted wave could be generated, when zero detuning from Brillouin frequency is satisfied, BDG process is polarization-decoupled BEFWM [31].

The difference between BDG and BEFWM are: in BEFWM the conjugate beam with high reflectivity due to the constant birefringence in two axes of the crystal in focal point, while FWM associated BDG is demonstrated under zero phase mismatches and zero detuning from Brillouin frequency. However the current application FWM enhanced SBS is focuses on quasi phase matching condition for acoustic wave generated by SBS random fiber laser, in which phase matching condition varies due to the time dependent laser gain via SBS process, and hence we need to study BDG frequency detuning at different location due to the laser gain depletion.

The equation governing the above mentioned process corresponds to the strong pump and weak Stokes and a weak probe beam. When the probe wave, which is the pulsed signal to detect reflection from the pump and Stokes wave that formed the random SBS lasing process, is weak comparing with pump beam and continuous Stokes beam due to the lower average power, we can simplify the FWM associated SBS process as following equations under phase matching condition [32]:

2a $$ \frac{d{E}_s}{dz}=-i{\eta}^{\ast }{\left|{E}_p\right|}^2{E}_s $$

2b $$ \frac{d{E}_{pr}}{dz}= i\eta {E}_p{E_s}^{\ast }{E}_d{e}^{-i\Delta kz} $$

2c $$ \frac{d{E}_d}{dz}=-i{\eta}^{\ast }{E_p}^{\ast }{E}_s{E}_{pr}{e}^{i\Delta kz} $$

2d $$ \eta =\frac{\pi^2{\gamma}_e^2}{\rho_0c{\lambda}_p^2{n}_x{V}_a\left({\Omega}_B-\Omega -\frac{\mathrm{i}{\Gamma}_B}{2}\right){A}_{eff}^{ao}}=\frac{g_p{\Gamma}_B}{4\left({\Omega}_B-\Omega -\frac{\mathrm{i}{\Gamma}_B}{2}\right){A}_{eff}^{ao}} $$

$$ \Delta k={k}_{pr}+{k}_d-{k}_p-{k}_s $$

$$ {A}_{eff}^{ao}={\left[\frac{\left\langle {F}^2\left(x,y\right)\right\rangle }{\left\langle {F}^2\left(x,y\right){F}_A\Big(x,y\Big)\right\rangle}\right]}^2\left\langle {F}_A^2\left(x,y\right)\right\rangle $$

Where *E*_*pr*_ is the field for the probe beam, *E*_*d*_ is field for diffracted beam, and *E*_*p*_ is the field for the pump beam, *F*(*x*, *y*) is the dimensionless fundamental mode profile optical wave. *g*_*p*_ is the Brillouin gain coefficient at pump wavelength, *n*_*x*_ is the refractive index of the PMF in fast-axis. ${A}_{eff}^{ao}$ is the acousto-optical effective area, *F*_*A*_(*x*, *y*) is the dimensionless mode profile of the acoustic wave (only fundamental acoustic mode is considered). The angular brackets 〈〉 denote averaging over the transverse cross section of the fiber. The detected beam in section 4 is |*E*_*d*_ (*z*)|^2^.

For detection of the diffracted wave, the phase-matching condition is required or nearly so, so that the Brillouin frequency along the two axes could be considered equal, since the frequencies of the pump wave and the probe wave have a difference determined by the birefringence of the PMF. The above assumptions result in a value of η with a percentage difference. The real part and the imaginary part of η are governed by Kramers–Kronig (K-K) relation associated with SBS amplification.

## BOTDA

### Basic principle of BOTDA system

**Fig. 1:**
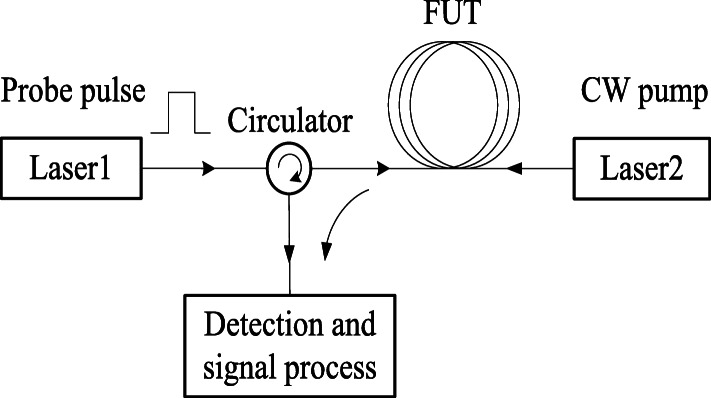
Schematic of the BOTDA by two laser and Brillouin spectrum

The distributed Brillouin sensor (DBS) has been developed [11, 21] for measuring the strain and temperature along the entire length of a sensing fiber [12]. BOTDA is one of the most well developed sensor techniques of distributed Brillouin sensing. The schematic principle of the BOTDA is illustrated in Fig. 1. The frequency difference between two lasers can be locked at an accuracy of Hz level at the different beat frequencies close to *ν*_*B*_. A simplified version can be achieved with a single laser, and Stokes wave with down or upper shifted Brillouin frequency for Brillouin gain or loss process [12]. The Brillouin interaction is localized in the time-domain by exploiting a pulsed pump (or probe) wave. An acoustic wave is generated locally at the point where the pulsed pump and the probe CW meet and interact. The spatial interaction confinement determined by the pulse duration τ defines the spatial resolution of the sensor as: $\Delta  z=\frac{1}{2}{V}_g\tau$, where *V*_*g*_ is the speed of light in the fiber and the factor 2 represents the round-trip propagation. The amplitude of the Brillouin interaction depends on the pump-probe frequency offset to the local BFS of the fiber section and their relative power ratio. The amplified probe wave propagates back to the photo-detector placed at the end of the fiber, where the time evolution of the intensity is detected. The time dependent probe wave power, often called *BOTDA trace,* can be directly converted to the distance-dependent information via $z=\frac{V_gt}{2}$. The pulse repetition rate is limited by the round trip time of pulses in the fiber to avoid trace overlapping between two pulses.

**Fig. 2:**
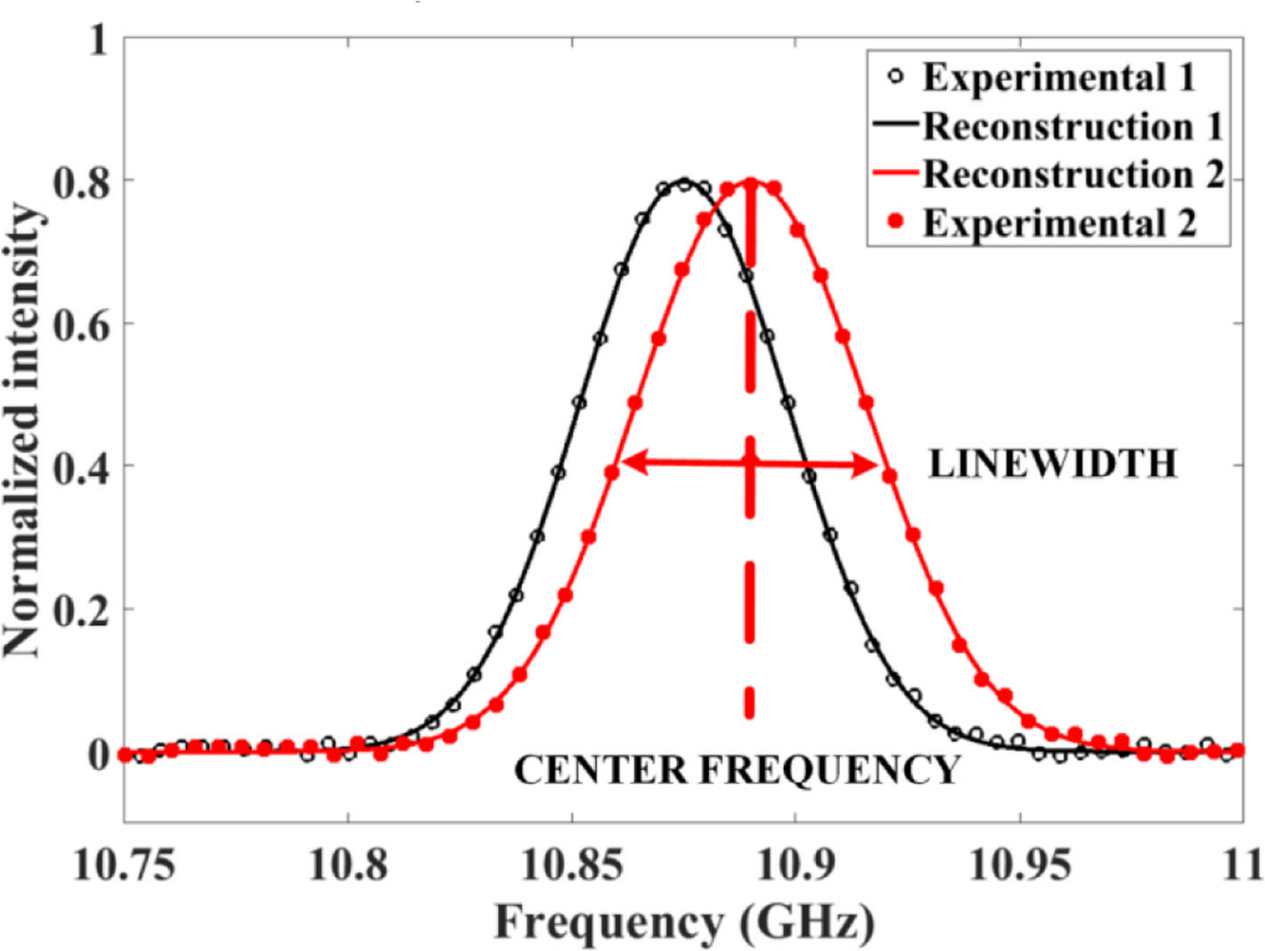
The Brillouin gain spectrum

After collecting the time-domain waveform at one beat frequency, the lasers are locked to a different beat frequency and another time-domain waveform is collected. This process is repeated for a range of laser beat frequencies such that the entire Brillouin spectrum is scanned over. The local BGS at each fiber section can be reconstructed by scanning the pump-probe frequency difference around the estimated BFS. The local BFS is determined by the fitting algorithm to the two adjacent measured local BGS at each fiber section, which is the pulse width associated with spatial length. An example of entire time-domain waveforms at different beat frequencies for Brillouin gain spectrum is shown in Fig. 2. With all of the time-domain waveforms collected, the Brillouin spectra can be obtained by displaying the data in the frequency-domain. For a given fiber location, a plot of Brillouin gain vs. laser beat frequency can be produced which shows the Brillouin spectrum of the fiber at a particular location. The entire data collection process will take several minutes depending on the range of frequencies scanned and the scanning step.

### Laser locking technique for high resolution BFS measurement

The frequency difference of the two lasers drifts with time around the Brillouin frequency, which increases the measurement uncertainty for BFS. To mitigate this effect, laser injection locking was proposed, which required the carrier laser (so called slave laser) frequency to be locked on one of the master laser side modes [33]. The spatial resolution of the distributed Brillouin sensor based on injection locking technique is on the order of meters, which is limited by the phonon lifetime. Although the injection locking method is simple and does not need stabilized lasers, it requires the locked frequency equals to one of the master laser side modes, which limits the frequency locking range. Moreover, the tuning of the laser frequency in the injection-locking was realized by changing the microwave generator frequency that was applied to the slave laser within the locking range, which limited the tuning range. In addition, the change of the tuning frequency had an impact on the laser (slave) power stability. In addition, a power change of the master laser would affect the master laser side modes and had a direct effect on the slave laser, and it takes time for the slave laser to re-establish the stabilized frequency condition after each tuning step. This limits the tuning speed and sensor response time.

Alternatively, one can use two laser sources and lock their difference via heterodyne through optical delay line to stabilize offset frequency at the Brillouin frequency for distributed sensor system. Because of external tuning of optical delay line [22], it allows wide tuning range and response time of less than 1 millisecond due to external tuning capability. Hence the tuning did not affect the laser power and the tuning and signal processing time was much shorter than the injection locking required tuning time. Furthermore, in order to make the sensor system simple and cost-effective, a hardware proportional-integral-derivative (PID) controller was used to lock the beat frequency that made the tuning of the laser frequency much faster, and BFS accuracy is in the range of kHz [22].

Figure 2 shows how the peak frequency of the Brillouin spectrum changes with local temperature for the pump laser frequency at 1550 nm in telecom fiber SMF28. The solid circles show the spectrum of the fiber at room temperature (23 °C), while the blank circles represent the spectrum of 1 m sensing fiber immersed in ice-water (0 °C). The solid and dashed lines are the results of data reconstruction [23]. It shows that the central frequency shifts by 27 MHz corresponding to the temperature change of 23 °C. The spectrum has no distortion and fits well with a Lorentzian shape.

### Trade-off between spatial resolution and measurement accuracy

The characteristics of the Stokes pulse have a significant impact on the performance of the DBS. The extinction ratio [34] and width [35] of the pulse have a direct impact on the spectral width of the detected Brillouin spectrum and thus the frequency resolution of the system and strain/temperature measurement accuracy [36]. There is a trade-off between the spatial resolution of the system and the measurement accuracy. In order to achieve high spatial resolution, the probe pulse should be as narrow as possible, however, as the pulse duration is reduced, the resulting spectrum of the pulse broadens. Since the measured Brillouin spectrum is a convolution of the pulse spectrum with the natural Brillouin spectrum [35], when a short pulse with a very broad spectrum is used, the measured Brillouin spectrum is also very broad.

**Fig. 3:**
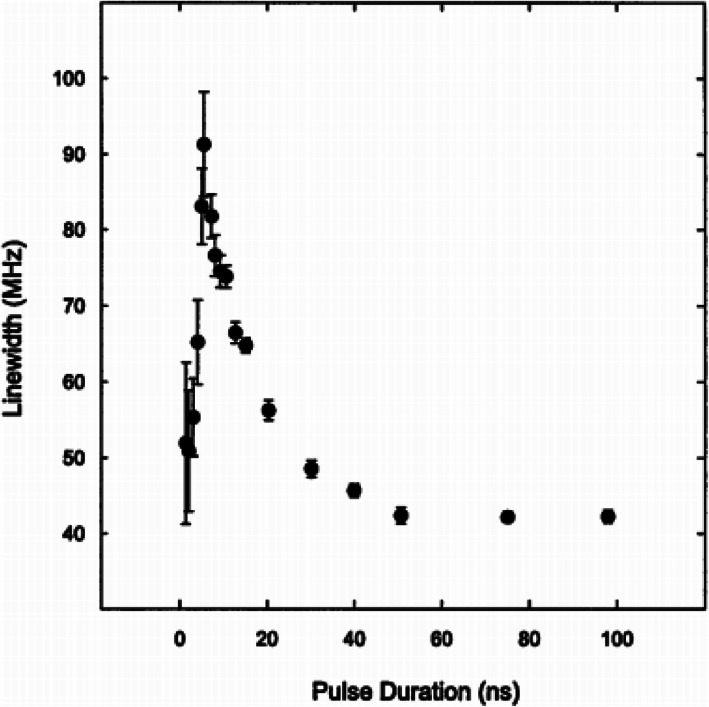
Brillouin linewidth as a function of pulse duration

One way to get around this trade-off between spatial resolution and measurement accuracy is to take advantage of the behavior observed by Bao et al. [37] that for very short pulse duration (shorter than the acoustic relaxation time of 10 ns), the Brillouin linewidth narrows back down to the natural Brillouin linewidth as shown in Fig. 3. This behavior has been explained as being caused by the DC component of the probe wave that is transmitted into the fiber due to the finite extinction ratio of the electro-optic modulator [38, 39]. When the acoustic wave produced by the DC base is roughly equivalent to that produced by the pulse, the measured Brillouin spectral width remains relatively narrow (due to the influence of the DC base), while a high spatial resolution of the short probe pulse is retained.

The first sub-meter spatial resolution in BOTDA was demonstrated in 1998 at 50 cm spatial resolution by Brown et al. [40], achieved by a compound spectrum analysis method. The DC portion acts as pre-pumping for short pulse-based BOTDA, which forms the foundation for the pre-pumping technique that have been proposed to realize centimetres spatial resolution including the dark-pulse technique [41], pulse pre-pump (PPP) technique [42], differential pulse-width (DPP) technique [4, 43, 44], Brillouin echo technique [45], Brillouin dynamic grating [46]. Ref [43] demonstrated 1 million sensing points for distributed Brillouin sensor which combined with long sensing length (10 km) and high spatial resolution (1 cm) based on DPP approach.

The high spatial resolution of 1 cm even 1 mm in real-time detection requires a very high sampling rate digitizer with broad bandwidth, which makes the entire sensing system to be very expensive. Alternatively, computation distributed sensing [47] offered a solution through the ghost imaging (GI) principle. Because distributed sensor receives the spatial information from backscattered optical signals in time-domain, the spatial/temporal “image” is transformable, which eliminates the need of continuous fast-speed acquisitions that can be realized by sending a pre-known binary pattern optical pulse to the spatial scattering object sequentially in time. The detector received scattered signals periodically with an acquisition rate inversely proportional to the total time duration of the light sequence. The correlation will be calculated between detected and pre-known binary patterns in time-domain to get “distributed image” [47]. The GI technique, with 3 orders of magnitude improvement in sampling rate, can reduce the cost of the BOTDA significantly. Note this cost-saving comes at the expense of large computation power and slower response time for overall detection speed.

### BOTDA for acoustic/impact wave detection, and dynamic strain measurement

**Fig. 4:**
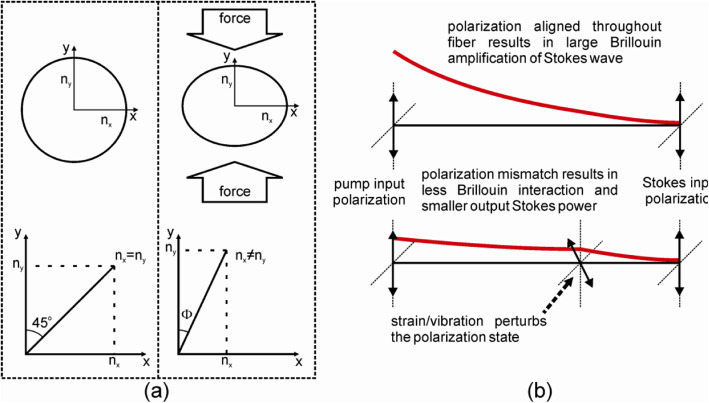
**a** Local birefringence change caused by force on optical fiber. **b** Sensing mechanism based on perturbation of the polarization states – the growth in the Stokes wave traveling from right to left is represented by the red line

In structural health monitoring, it is often advantageous to monitor the dynamic behavior of a structure in real-time. The traditional distributed Brillouin sensor does not allow for this dynamic measurement due to the need to sweep the frequency difference between the two lasers and subsequent averaging of waveforms. A real-time vibration sensor based on polarization-state perturbations in stimulated Brillouin scattering instead of resonant frequency mismatching monitoring of the Brillouin spectrum can achieve distributed impact/acoustic wave in 2008 [48]. The long measurement time of traditional distributed Brillouin sensors is avoided by eliminating the frequency sweep of the pump and Stokes lasers and instead of locking them at a single beat frequency corresponding to the static strain of the structure in which the fiber is embedded. Note, the acoustic wave often has a small displacement of sub-micrometers in length, such a small change is associated with the Brillouin frequency shift of tens of kHz, which is hard to detected by fitting the Brillouin spectrum to get BFS method [49]. However, the peak Brillouin gain varies with the state of polarization (SOP) of pump and Stokes wave, when a sound wave (pressure) is propagated through a fiber location in SMF, it changes the local SOP with the time period of acoustic wave, and hence the peak Brillouin gain changes accordingly at different fiber location as illustrated in Fig. 4. As a result, it enabled distributed impact wave detection based on peak Brillouin gain. This unique sensor allows measurement of vibration frequencies along a sensing fiber as shown in laboratory experiments and also the detection of impact waves from passing vehicles in field tests in which the sensor was embedded in the concrete pavement of a highway.

Most recently, more works have been developed around the acquisition of BGS to save sweep time for ultrafast strain measurement. Here, we introduce three methods for fast BOTDA sensing: (1), Frequency comb-based sweep-free method proposed in 2011 [50]. It is based on the Brillouin interaction between two combs of probe and pump waves. Instead of frequency scanning, multi-tone probe waves simultaneously are detected from the fiber length with an equal number of pump pulses of various frequencies, so that each pair of probe and pump waves scan at a different fiber location for dynamic strain measurement. However, the frequency interval for the comb limited the spatial resolution to tens of meters. (2), Slope-assisted method: It skips the BGS scanning process by taking the advantage of the linearity at the edge of the BFS to convert the BFS shift due to the strain change to the amplitude variation [51], so that a single pump pulse can demodulate the distributed BFS along the fiber. Note the sampling rate is only limited by the length of the fiber without averaging, but the dynamic range is restricted by the linewidth of the BGS [52]. (3), Fast-frequency sweeping method: It is based on compressing frequency switching time for the probe wave to increase the speed. It provides the minimum time duration for a BGS scan based on the fast switching by an arbitrary waveform generator (AWG) instead of a synthesizer based electronic sweeping time. The measurement procedure is similar to normal BOTDA [53]. For certain sensing fiber length, the required time for single BGS scanning could be reduced to sub-ms. The modified version is based on an optical chirp chain, named OCC–BOTDA, where the probe wave is frequency modulated into short optical chirp segments and then cascaded into an OCC. Only a single-shot pump pulse is required to recover the distributed BGS along with the OCC probe wave over wide frequency range. The maximum sampling rate can reach MHz, limited by the length of the fiber [54]. The mechanical shock wave can be detected for MHz event in dynamic strain measurements of periodic mechanical vibration.

### Distributed hydrostatic pressure measurement

Detection of unintended inner obstacles or leak of a pipeline is an important subject for oil and gas industry, one solution is to monitor the variation of local pressure in a distributed method. The pressure change and BFS has a linear relation, which makes it possible to detect BFS with BOTDR for distributed pressure measurement using special coating [55]. In field, pressure changes with temperature, it is important to monitor two variables in order to increase resolution of the pressure measurement using BOTDA approach [56]. The introduction of the Brillouin dynamic grating in PMF has enabled pressure sensitivity to be improved by a factor 60–160 times comparing with direct BFS measurement using special coated SMF [57]. Using polarization-maintaining photonic crystal fiber based on Brillouin dynamic gratings, the temperature compensated distributed hydrostatic pressure sensor can be achieved with 0.03Mpa with 20 cm spatial resolution [58].

## BOTDR

### Basic principle of BOTDR

**Fig. 5:**
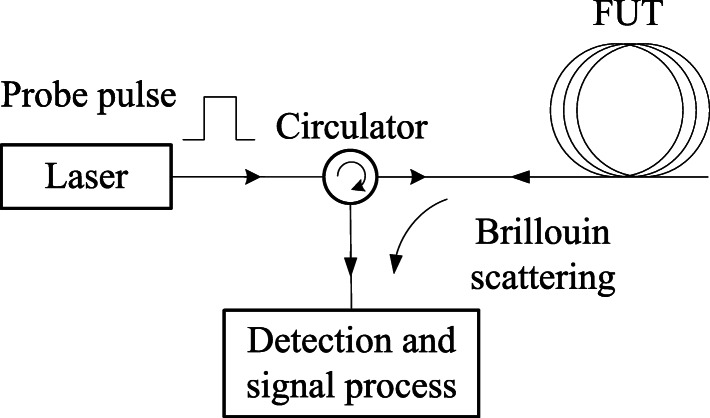
Schematic diagram of BOTDR

As long as the input light is scattered without strongly altering the properties of the medium, then the scattering would be described as spontaneous. When a pulsed signal is sent to the fiber, through the spontaneous Brillouin scattering (SpBS) one can realize a distributed sensor based on back-scattered signal via Brillouin optical time-domain reflectrometry (BOTDR), which has the advantage of one-end access compared to BOTDA system, and it is very attractive for the field tests, especially when the sensing fiber is broken and the breaking point could be measured. This technique has been used in many fields monitoring of civil structures. Because of the weak spontaneous Brillouin scattering, the spatial resolution and strain resolution are lower than the SBS based BOTDA sensor. The setup of BOTDR is shown in Fig. 5.

The spatial resolution in BOTDR is also confined by the pump pulse duration, $\Delta  z=\frac{1}{2}{V}_g\tau$. Since the amplitude of SpBS is much lower than that in SBS, the back scattered signal power is much weaker. To enhance detection sensitivity, coherent detection with a strong optical or electrical local oscillator is used [12, 59]. The pump-probe scanning for BGS reconstruction is conducted in the electrical domain with the scanning of the electrical local oscillator. The disadvantages of the weak SpBS signal are the impacts of the undesired Rayleigh back scattering and the Fresnel reflection from the connectors and at the end of the fiber.

### BOTDR for dynamic strain and vibration measurement

Sloped assisted Brillouin gain can also be used in BOTDR, which allows dynamic strain measurement via one-end access sensing fiber [60]. It was demonstrated at 7.6 Hz sampling rate, the pipeline vibration was measured with 12 mm displacement over 1 m spatial resolution. The short-time Fourier transform was introduced to BOTDR with small gain SBS amplification, which allows 60 Hz vibration detection over 6 m fiber section [61].

### Distributed pressure sensing based on BOTDR

The motivation of distributed pressure sensing is the need to detect the physical location of a blockage or obstruction along the total length of a hydrocarbon pipeline or oil well [55]. The measurement of distributed hydrostatic pressure based on a specialty optical fiber with a pressure-enhancing coating, such as Teflon combined with a BOTDR system. The choice of combined Nylon and silicone is that it can lead to a maximum strain amplification of 30 times, which resulted on a minimum detectable pressure of 15 psi, which is equivalent to 0.345 MHZ in BFS.

## Distributed acoustic wave detection based on SBS random laser

The Brillouin dynamic grating was first observed by Song et al. [15] in a polarization-maintaining fiber (PMF). It was formed by the stimulated Brillouin scattering excited acoustic wave via a high power pulsed beam in one axis of PMF, and being detected by a weaker probe beam in another axis of PMF, the pulsed probe beam is reflected at a different wavelength with up or down conversion of the wavelength shift satisfied with the phase-matching condition which is related to the refractive index difference between the fast and slow axes. Such a process is equivalent to up or down conversion of the SBS depending governed by four wave mixing process.

### FWM enhanced SBS

The SBS effects are sensitive to the polarization states of the pump wave and Stokes wave. The Stokes wave gets the maximum gain from the pump wave when their polarization states are aligned. In contrast, if the Stokes wave is injected into the axis of PMF orthogonal to the polarization of pump wave, the SBS gain is negligible. In the PMF, the polarization states of the light can be decomposed linearly according to the direction of the slow and fast axis. However, the acoustic wave accompanied with the SBS effects is a longitudinal fiber density variation that is insensitive to the polarization direction. Therefore, the periodically modulated refractive index change of the fiber induced by the linearly polarized pump light in one axis can also modulate the phase of light in another axis. When the phase matching condition is satisfied, the two pump waves in the slow (fast) axis and two Stokes waves in the fast (slow) axis are coupled together through the same acoustic wave in the fiber. This coupling process is a four wave mixing (FWM) process, which can further enhance the SBS effects in the fiber [32]. The wavelength difference of two pump waves in slow (fast) axis is slightly different, which is determined by the birefringence of the PMF under the phase matching condition. Those kinds of FWM enhanced SBS have been studied in PMFs previously to realize high spatial resolution distributed sensing [62], to completely discriminate temperature and strain for Brillouin based sensors [63], to achieve tunable optical delays [64] and to measure distributed birefringence of PMFs [65]. Recently, a narrow linewidth acoustic wave was generated and detected through the FWM enhanced SBS in random SBS lasers [16]. This technique can help us to understand the fundamental physics of the lasing mechanism in random fiber lasers.

### Narrow linewidth acoustic wave generation and detection

**Fig. 6:**
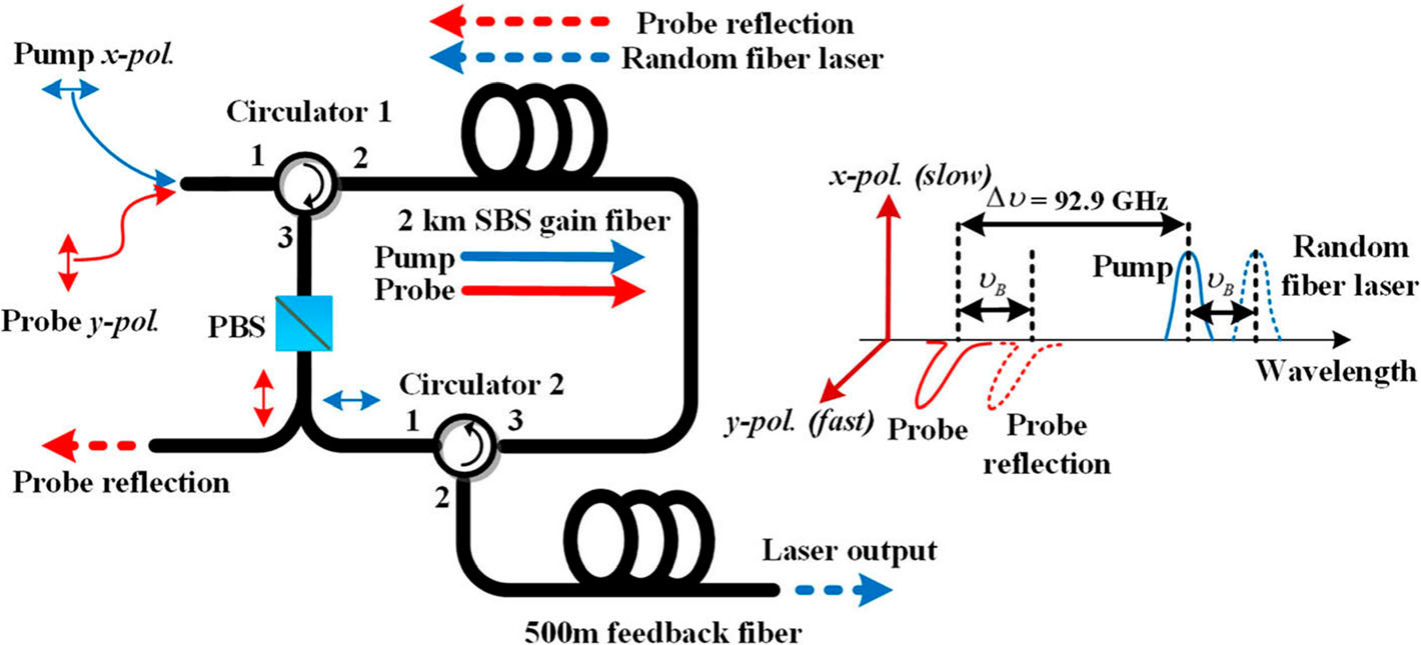
Schematic diagram of the operation principle of acoustic wave detection in the random SBS laser

The process of SBS is well known as a nonlinear interaction among the pump and Stokes fields and an acoustic wave. The properties of the pump fields and Stokes fields can be easily detected by the optical spectrum analyzer (OSA) and oscilloscope. However, the properties of the acoustic wave have been ignored for a long time. It is important to study the properties of the acoustic wave in optical process with narrow linewidth generation, for instance, in random SBS lasers. The random SBS lasers, which is one kind of random fiber laser based on Brillouin gain and Rayleigh scattering feedback, are demonstrated to have good performance in generating highly coherent photons [66, 67]. In order to study the properties of the acoustic wave in the random SBS laser, the FWM enhanced SBS process was employed to detect the properties of acoustic wave. The basic principle of the acoustic wave detection in the random SBS laser is shown in Fig. 6. In *x* polarization, the pump light with linear polarization is injected into the slow axis of the 2 km PM SBS gain fiber. The backward Stokes light passes through circulator 1, PBS, circulator 2 successively and then experiences the distributed Rayleigh scattering along 500 m PMF, providing feedback for random laser’s radiation. In *y* polarization, a probe light is also launched to the SBS gain fiber. When the wavelength difference between the probe light and pump light satisfies the phase matching condition, the probe light experience strong reflection by the acoustic wave induced Brillouin dynamic grating. In the experiment, the wavelength difference is determined by the birefringence of the fiber under phase-matching condition, for instance, the difference is around 92.9 GHz when the birefringence of the PMF is around 6.94 ×10^−4^. Because the dynamic refractive index change introduced by the acoustic wave in the fiber periodically modulate the phase of the probe light, the reflected probe light experiences a frequency shift with the same value as the frequency of the acoustic wave. The reflected probe light carries the properties of the acoustic wave, such as the phase noise, linewidth, intensity, which can then be demodulated by measuring the properties of the reflected probe light.

**Fig. 7:**
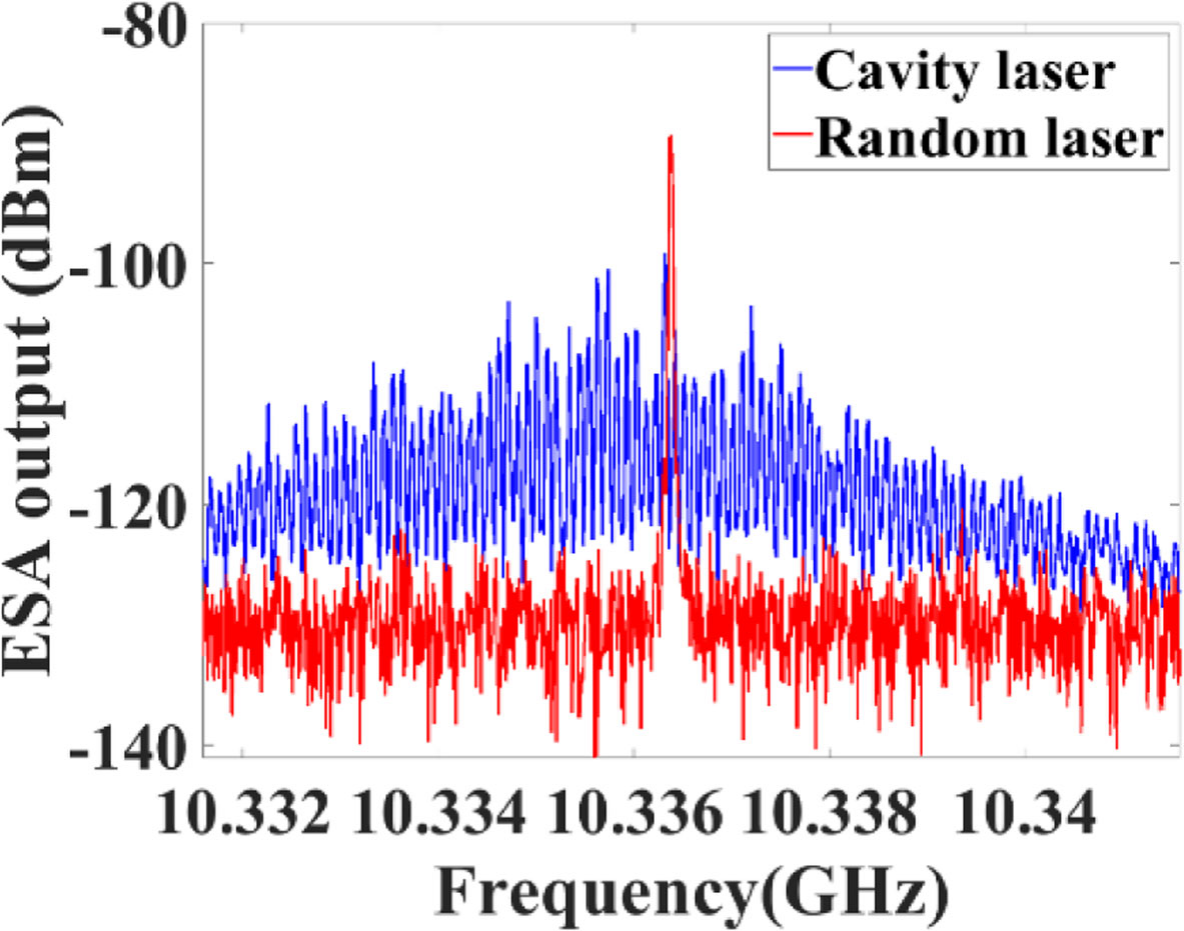
Mode structure of the acoustic wave in cavity SBS laser and random SBS laser

The random SBS lasers have advantages in generating narrow linewidth lasers due to their intrinsic narrow gain bandwidth. In [68], up to 10 Hz narrow linewidth lasers are generated in a random SBS laser. The narrow linewidth random SBS laser can be used in ultrasound sensing, narrow linewidth signal characterization and microwave generation. In [16], it is demonstrated that the spectra of the acoustic wave in the gain fiber are determined by the spectral convolution of pump light and its Stokes light. Therefore, considering the narrow linewidth property of the random SBS laser, if a relative narrow linewidth laser is used as the pump source, the linewidth of the acoustic wave also possesses the narrow linewidth property. In the conventional cavity Brillouin fiber laser, the acoustic wave possesses multimode structures, as shown in the blue curve in Fig. 7. In contrast, in a random SBS laser, the distributed feedback enabled by coherent Rayleigh scattering forms a “modeless” random distributed cavity. The randomly distributed cavity always selects a narrow lasing spectral peak at the highest gain, leading to that the frequency of the acoustic wave can be precisely aligned to the Brillouin frequency shift of the Brillouin gain fiber. Therefore, the red curve in Fig. 7 shows that the spectra of the acoustic wave stimulated in the random SBS laser have a single narrow linewidth peak. The distributed information of the acoustic wave can be measured by sending a series of probe pulses and measuring the time-resolved traces on the oscilloscope. Depending on the measurement time of one detection process, the distributed detection can be divided into static measurement and dynamic measurement.

### Static properties of the Brillouin dynamic grating in random SBS laser

**Fig. 8:**
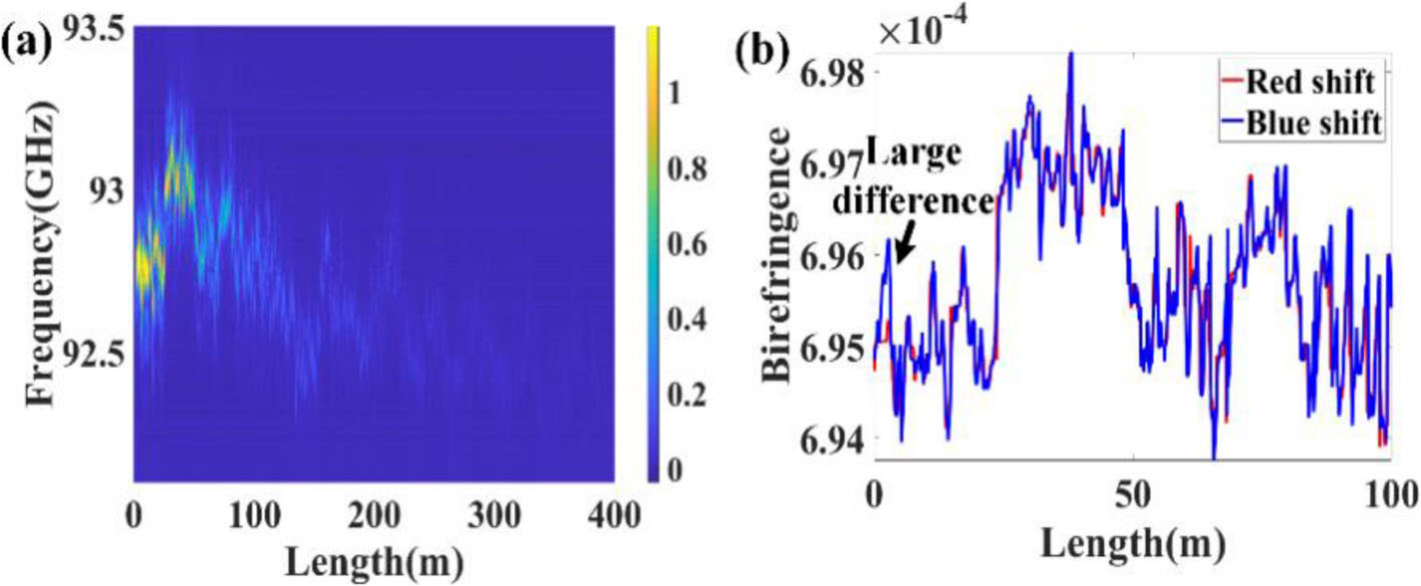
**a** Distributed reflection spectra of the Brillouin dynamic grating; **b** Birefringence of the Brillouin gain fiber measured by probe light red shift and blue shift when random SBS laser is in operation

In the static measurement, the frequency of the pulsed probe light is swept near the central wavelength of the acoustic wave induced Brillouin dynamic grating like the BOTDA technique. The frequency scanning process usually takes several minutes, so the static measurement characterizes the time-averaged property of the Brillouin dynamic grating. The spectra of the Brillouin dynamic grating reflect the distributed birefringence of the Brillouin gain fiber, as shown in Fig. 8. The reflectivity of the dynamic grating is determined by the refractive index modulation depth (intensity of the acoustic wave) of the fiber, which is proportional to the Brillouin gain of the random SBS laser. Because the Brillouin gain depletes exponentially in the spatial domain, the reflectivity of the Brillouin dynamic grating near the pump end is strong, while far away from the pump end it is very weak and barely can be detected. Different from the traditional fiber Bragg grating (FBG), the Brillouin dynamic grating is moving in the sound velocity in the fiber, so the reflected probe light experiences a Doppler frequency shift compared to the original probe light. Depending on whether the probe light co-propagate or counter-propagate with the Brillouin dynamic grating, the reflected probe light either experiences a Doppler red frequency shift or Doppler blue frequency shift. By comparing the birefringence difference measured by the two different methods, the SBS-induced birefringence change is measured to be 10^−7^ to 10^−6^ in the random SBS laser in [17].

### Dynamic properties of the Brillouin dynamic grating in random SBS laser

**Fig. 9:**
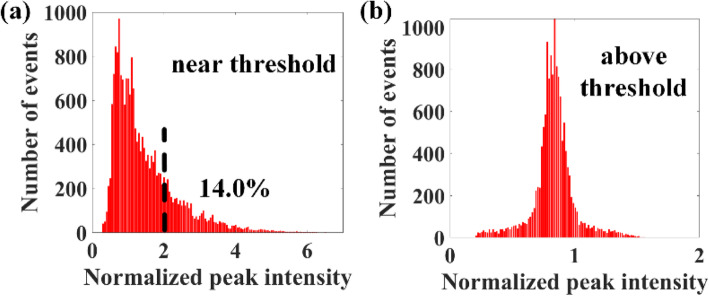
Histograms of the temporal intensity statistical distribution of the acoustic wave **a** near the random laser threshold; **b** above the random laser threshold. The x axis is normalized by the significant wave height

In the dynamic measurement, a series of pulsed probe light with a high repetition rate at fixed optical frequency under phase-matching condition is launched to the Brillouin gain fiber, thus the fast change of the reflectivity of the Brillouin dynamic grating is obtained. Due to the mode hopping of the random SBS laser, the reflectivity of the Brillouin dynamic grating varies in the time-domain. Near the lasing threshold, the histogram of the reflectivity variation shows L-shaped long-tailed characteristics of extreme value behavior at all positions of the Brillouin gain fiber as show in Fig. 9. The generation of an optical rogue wave, which is defined as the event of the peak amplitude more than twice the significant wave height, is detected in the random SBS laser. The significant wave height refers to the mean height of the highest third of the wave intensity. The L-shaped long-tailed characteristics reflect the stochastics intensity behaviour that is attributed by thermal process of an acoustic phonon with totally uncorrelated phase relation. In contrast, the statistical property of the intensity fluctuation above the lasing threshold is modified to Gaussian probability distribution owing to the stable establishment of the random laser emission. The dynamic properties of the Brillouin dynamic grating reflect the SBS dynamics, which is governed by the phonon lifetime in silica fibers. In the presence of the distributed optical feedback introduced by the Rayleigh scattering, the random feedback destabilizes relaxation oscillations in normal fiber lasers and lead to irregular fluctuations. The dynamic characterization method of the random SBS laser enabled by the FWM enhanced SBS process provides a new tool for understanding and exploring the fundamental physics of the lasing mechanism in random fiber lasers.

## Sensing parameters

### Spatial resolution

A distributed Brillouin sensor with a high spatial resolution is able to measure the physical parameter change more accurately and locate it in a very small scale, such as a crack and deformation in bridge, pipeline, this is particular true for the stimulated Brillouin scattering based BOTDA sensors. There is a trade-off between the spatial resolution of sensors and the measurement accuracy. In order to achieve high spatial resolution, the probe pulse should be as narrow as possible (spatial resolution of 1 m corresponds to a pulse duration of 10 ns). However, as the pulse duration is reduced, the resulting spectrum of the pulse broadens. Since the measured Brillouin spectrum is a convolution of the pulse spectrum with the natural Brillouin spectrum, when a short pulse with a very broad spectrum is used, the measured Brillouin spectrum is also very broad. One solution is to increase the weight of excitation of long phonons as discussed in BOTDA section to allow 1 cm spatial resolution [43]. Another method called double-pulse technique is used in reference [69] to improve the spatial resolution up to 20 cm. In reference [70], 20 cm spatial resolution also achieved by combining time-domain and correlation-domain analysis.

### Measurement resolution

The resolution is characterized by the standard deviation of the BFS value after many trace averaging (usually 1000s). A high Brillouin gain, low noise level, or enhanced signal-to-noise ratio (SNR) by a nonlinear optical signal processing can lead to a sharp spectrum profile [71]. The resolution is not a constant in the entire fiber length, but higher at the pump launching end and lower at the probe launching end of the fiber, respectively. The improvement of the measurement accuracy can be achieved by coding the pulse with longer processing time [12].

### Sensing range

BOTDA offers the best performance for the long-range measurement applications which are the main market drivers for monitoring large-scale structures such as power cables, railways, and oil and gas pipelines. Long fiber lengths introduce high attenuation, which can be compensated by high pump power to improve signal to noise ratio. However, the increased powers are constrained by nonlinear optics effect, such as modulation instability [72], self-phase modulation [73], and probe power is limited by the threshold of the SBS, which will reduce effective sensing length [74]. In addition, the nonlocal effect is another detrimental effect for long-range BOTDA or BOTDR. The nonlocal effect is caused by the accumulated interaction between the pump pulse and probe wave, which distorts the Brillouin spectrum and subsequently introduces fitting errors for Brillouin frequency shift determination [75]. To achieve long-range Brillouin sensing, the nonlocal effect should be mitigated. The nonlocal effect is introduced during the frequency scanning process in the Brillouin gain or loss scheme. The power transfer between the pump and probe is high near the peak of the Brillouin frequency shift (BFS), while it is low away from the fiber BFS, and this may cause a fluctuation on the pump pulse power that can distort the Brillouin spectrum, especially in the far end of the sensing fiber. Time-division [76] and frequency-division [77] BOTDA systems can reduce the Brillouin interaction length, and hence mitigate the nonlocal effect, as the Brillouin interaction is constrained within counter-propagated double pulses which moved in time with multiple pulses at different time, although it requires longer measuring time due to many long probe pulses are needed to cover the entire sensing length. Similarly using combined fibers of different Brillouin frequencies with different lengths have effectively separated total sensing range to many different segments with larger scanning Brillouin frequency range, which reduced nonlocal effect significantly. BOTDA based on a frequency modulated probe wave was demonstrated to be free from the Brillouin threshold limitation and detrimental nonlocal effect, which achieved the 100 km sensing distance with 1 MHz BFS accuracy [78].

To increase the sensing range of BOTDA sensors, several technologies have been proposed: (1) To locate the optical repeaters constituted by the Erbium-doped fiber amplifiers (EDFA) at critical positions over the sensing distance for 150 km sensing range [77]. The EDFA can also be placed in front of the detector to pre-amplify the Brillouin signal before detection which enabled 120 km [79]. (2) Distributed Raman [80] or Brillouin amplifier [81] offered distributed gain to compensate fiber loss for 100 km range. (3) Optical pulse coding [82] is an effective method to increase the SNR and extend the sensing distance to the order of 100 km. (4) propagation loss compensation, tailored compensation is utilized for the propagation loss of the pump pulse and is demonstrated for long-range and high-resolution distributed sensing, up to 51.2 km sensing range with spatial resolution of 20 cm [83]. (5) Optimizing Image Denoising methods including linear and nonlinear image processing methods for BOTDA data denoising are presented in [84], and 100 km sensing range with 2 m spatial resolution is achieved.

For long-range applications, the response time is an issue, especially for the small change detection which requires action to prevent bigger problems. However, current long range BOTDA sensors require frequency mapping of the Brillouin spectrum and a large number of average times, which is time-consuming for the end-user applications. A fast long-range BOTDA based on the optical chirp chain (OCC) probe wave and Brillouin loss system can solve the slow response problem [85]. The frequency-sweeping span of the microwave short-chirp segment covers several hundred MHz in tens of nanoseconds, which can be considered as the time compression of the modified microwave signal for dynamic sensing in BOTDA sensors over 150 km. The comparison of the proposed BOTDA schemes in terms of spatial resolution, sensing accuracy and sensing range is shown in Table 1.

**Table 1:** Comparison of the proposed BOTDA schemes in this work

	[40] compound spectrum analysis method in 1998	[41] Dark pulse in 2005	[42] Ppp method in 2005	[44] Dpp method in 2009	[45] Brillouin echo technique in 2010	[83] tailored compensation in 2017	[84] Image Denoising methods in 2018
Spatial Resolution	50 cm	5 cm	10 cm	10 cm	5 cm	20 cm	2 m
Sensing Accuracy	40 μɛ	±20 μɛ	25 μɛ	16 μɛ	60 μɛ	29 μɛ	20 μɛ
Sensing Range	100 m	100 m	kilometers	12 km	5 km	51.2 km	100 km

### Measurement time and sensing response

The measurement time of a BOTDA sensor is in the range of a few to tens of minutes, which is attributed to the need to scanning the Brillouin gain spectrum, and many numbers of BOTDA trace averaging to bring better spatial and sensing resolution.

There are three main factors that limit the measurement speed [53], (a) the time of flight, also called round-trip time ${T}_{round}=\frac{2 nL}{c}$; (b) number of averaging *N*_*avg*_, to achieve the required signal to noise ratio (SNR); (c)scanning step *M*_*freq*_, *N*_*freq*_ is the number of steps for BGS. The overall time required for a complete BGS spectrum is given by

$$ {T}_{BGS}={N}_{avg}{N}_{freq}{T}_{round} $$

There are a few approaches that have been proposed to reduce *T*_*BGS*_, as explained in section 3.1. (1): sending large frequency components by a large pulse in sub-ms time frame [53]. (2) The probe wave is a multi-tone frequency being sent simultaneously to fiber [86], which reduces the need of scanning frequency. Also, in reference [87], the dual-polarization probe with orthogonal frequency-division multiplexing (OFDM) modulation is used to acquire the distributed Brillouin gain spectra, the sensing speed is only limited by the length of fiber. (3) slope-assisted (SA) BOTDA, which eliminates the BGS scanning by converts BFS measurement into intensity measurement at the maximum slope (quadrature point) of BGS [51, 52]. Currently, SA-BOTDA is one of the popular techniques for dynamic sensing. A summary table illustrates the details of the proposed fast BOTDA as shown in Table 2.

**Table 2:** Comparison of the proposed fast BOTDA schemes in this work

	[53] optical frequency-agile technique	[52] slope-assisted (SA) BOTDA	[50] Frequency comb-based sweep-free method	[54] OCC–BOTDA
Sampling Rate	~10 kHz	~ 100 Hz	~ 33 kHz	6.25 MHz
Sensing Accuracy	~ 5 μɛ	n/a	29 MHz	35 μɛ (with 30 points moving average)
Sensing Range	100 m	85 m	2 km	1 km

## Limitations in the distributed Brillouin sensors

The Brillouin frequency (associated with strain and temperature) is determined by finding the center frequency of the Brillouin gain or loss spectrum. This is obtained by fitting a calculated curve to the measured data at different locations over a large number of spatially resolved points within spatial resolution. The accuracy of this determination decreased with reduced SNR, a decrease in the number of acquired BOTDA or BOTDR traces in scanning Brillouin spectrum steps, an increase in linewidth of the lasers, and systematic deviation of the spectrum from its theoretical shape. The four effects can reduce the strain or temperature resolution as one improves the spatial resolution. Considering the loss of signal due to increasing the fiber length from fiber attenuation, there is a three-way trade-off for (1) spatial and (2) strain/temperature resolution, and (3) total sensing length.

Furthermore, increased signal averaging leads to more time-domain waveforms with higher SNR, which however, leads much longer measuring time due to extended acquisition time. Therefore, an analysis of the performance of the sensor system needs to consider acquisition time as well as the other three system parameters. The analytic expression of the Brillouin frequency shift error *δν*_*B*_ can be expressed by fitting Brillouin gain or loss spectrum [67].

$$ \delta {\nu}_B\approx \frac{1}{SNR(z)}\sqrt{\frac{3}{4}\updelta {\gamma}_B} $$

where *γ*_*B*_ is the full width at half maximum (FWHM) of the Brillouin gain spectrum, $SNR(z)=\frac{1}{\sigma (z)},\sigma (z)$ is a root mean square (RMS) value of the noise of the measured Brillouin gain spectrum at position *z.*

- Digitizer noise: Although the level of noise in the system is largely constant as the noise floor of the receiver, the signal level is not, and is expected to decrease as the spatial resolution is shortened, and hence it leads to reduced SNR. Averaging of N samples increases the SNR by a factor of at the expense of extending the acquisition time, which includes the coded pulse technique. The maximum SNR possible for a 16-bit sample is about 51 dB but typical experimental SNRs are limited to about 40 dB, which is due to systematic deviation of the spectra from the fitted shapes. For very high spatial resolution, such as 1 mm, the signal level is likely 1/10 of full scale of the digitizer, which makes quantizing noise as an issue.
- Effect of SNR: the main noise source of a BOTDA system is the detector, that is, the sensing system noise is mainly composed of thermal noise, shot noise, and the relative intensity noise (RIN) in the laser source, as well as amplified spontaneous emission (ASE) noise from the EDFA, which gives the photo-current noise of beat between signal and spontaneous noise. In [88], a novel configuration is proposed, which could eliminate many intensity related noises in BOTDA system.
- The extinction ratio of the optical pulse and pulse height fluctuation: In order to produce the pulsed probe beam, a Mach-Zehnder electro-optic modulator (EOM) is used to turn the probe beam on and off to produce the spatial resolution for distributed sensing. It is crucial to keep the pulse energy uniform for constant signal to noise ratio. However, the variable DC leakage (pulse base) through the EOM should be locked. The EOM can never block 100% of the light from the laser and so the probe beam will always have a DC component in addition to the pulse. The critical problem with the EOM pulsing system is that, over time, the DC component does not stay constant but slowly drifts resulting in reduced sensor performance – even when a stabilized voltage is applied. This means pulse energy fluctuates with time, causing SNR variation and higher measurement error. To reduce the drift of the pulse base, one can lock the pulse base using a lock-in amplifier; and to set value using a proportional-integral-derivative (PID) control algorithm to lock the base [88].
- Effect of fibre birefringence: the position dependent fibre birefringence also contributed the similar level of uncertainty [12], such effect can be mitigated by polarization divinity detection or using polarization scrambling to reduce polarization dependence of the Brillouin gain at various fibre locations.

## Sensing with specialty fibers for simultaneous temperature and strain measurement

The motivations of SBS in specialty fiber in this section are (1) to demonstrate simultaneous temperature and strain sensing with a spatial resolution of centimeters; (2) to provide specific sensing parameters that are not available with telecom SMF (SMF28). Since temperature and strain both cause changes in the Brillouin frequency, it is impossible to separate their effects without additional information, unless two fibers are used with one for temperature isolated from strain and other fiber is subjected to both temperature and strain, demonstrated by Bao et al. in 1994 [89]. One can measure two variables to recover the temperature *T* and strain *ε* simultaneously. The sensor system can be characterized by the matrix equation for T and ε measurement,

$$ \left[\begin{array}{c}\Delta  {\upsilon}_{B1}\\ {}\Delta {\upsilon}_{B2}\end{array}\right]=\left(\begin{array}{cc}{C}_{T1}& {C}_{\varepsilon 1}\\ {}{C}_{T2}& {C}_{\varepsilon 2}\end{array}\right)\left[\begin{array}{c}\Delta  T\\ {}\Delta \upvarepsilon \end{array}\right] $$

Where *C*_*i*_ are the thermal and strain coefficients for the Brillouin frequency shifts. Δ*υ*_*Bi*_ are the measured BFS with multiple Brillouin peaks relative to the reference temperature and strain change. The power and linewidth change of the Brillouin spectrum can also be represented by the above equation to replace the BFS change in above equation. Note the C parameter should differ significantly to achieve the required sensitivity. To get 2nd parameter, one can simultaneously measure temperature/strain and vibration over 150 km, is elaborately designed via integrating the Brillouin optical time-domain analyzer (BOTDA) and phase-sensitive optical time-domain reflectometry (Ф-OTDR) [90] or hybrid Raman/BOTDA sensor system [91]. Another method is to obtain multiple peaks of the Brillouin scattering which means we need to enhance higher order acoustic modes in SMF [92]. SBS in optical fibers is due to the interaction of the laser beam with different acoustic modes supported by the single mode fibers with complex index profiles, such as LEAF fiber which presented four Brillouin peaks even though it is SMF. Because of the solid-state nature of the glass materials, these modes are defined by the spatial distribution of the longitudinal and shear velocity and density of the core and cladding material, which are associated with thermo-mechanical response and scattering efficiency of the Brillouin process. To create high order acoustic modes for multiple Brillouin peaks [92], when fibers operate close to the cutoff of higher-order acoustic modes at the saturation regime of pump–probe Brillouin scattering, when the pump is depleted by interaction with fundamental acoustic mode, it offers the maximum ratio of higher-order acoustic and optical interaction peaks relative to the main Brillouin peak. Ideally, fiber’s acoustic velocity and refractive index profiles should be tailored to enhance the gain on interaction of one or more acoustic and optical modes. Lee et al. demonstrated the interaction of optical and multiple acoustic modes [93] for temperature and strain measurement using dispersion shifted fiber in 2001 with meters spatial resolution through scanning Brillouin spectrum via BOTDA. Alternatively one can also measure two beat frequencies among different Brillouin peaks in LEAF to realize temperature compensated strain measurement [94].

Another example is photonic crystal fiber (PCF) with multiple Brillouin speaks [95], BGS in PCF composed with one main peak shape with 2nd peak superimposed with three additional peaks. The multi-peaked structure was attributed to several groups of guided acoustic modes, each with different proportions of longitudinal and shears strain in the core [96]. Luckily, the two peaks have distinct temperature and strain coefficients for the BFS [97] attributed by the identical coefficients of SMF 28 and a new parameter likely contributed by combination of guided acoustic modes in the core. This feature enabled the first distributed temperature and strain sensing in PCF with BOTDA with spatial resolution of 15 cm using BOTDA sensor in 2004 at wavelength of 1319 nm. The temperature-compensated distributed hydrostatic pressure sensor was also demonstrated in polarization maintaining (PM) PCF based on Brillouin dynamic gratings [58]. The same idea can be applied to a few mode fiber [98] for two lowest modes of *LP*_01_, *LP*_11_ to realize the discrimination of temperature and strain.

As long as the thermo-mechanical response is related to the interaction of the scattered optical mode with the guided (or anti-guided) acoustic wave, both the optical and acoustic properties of the fibers may be functions of the environmental changed parameters. The acoustic-optic interactions in such fibers involve optical and acoustic modes that have the most similar shape with the largest spatial overlap [99], as demonstrated in [100] with analysis involving modes possessing orbital angular momentum, while Wu et al. [101] described a hybrid Raman-Brillouin system in a few-mode fiber.

The stress rod in polarization maintaining fiber (PMF) with different thermal coefficient leads to temperature and strain dependence in the Brillouin gain and linewidth, which has little dependence in telecom fiber (SMF 28), as a result, they can use to make strain and temperature sensing in addition to Brillouin frequency shift with single Brillouin peak in Panda and Bow tie PMF [102]. The simultaneous measurement of Brillouin frequency and changes in birefringence in a polarization-maintaining fiber with two parameters allowed temperature and strain sensing [29, 30, 103]. The use of Brillouin scattering coupled with thermally-induced changes to the emission spectrum of Er-doped into the fiber provides another solution for simultaneous temperature and strain sensing [104].

The multi-core fibers also offered the solution. For example, Li et al. proposed and demonstrated a dual-core fiber for discriminative sensing [105]. In this case, the two cores have non-overlapping values of BFS and have differing thermal and strain coefficients. Mizuno et al. [106] described the seven-core fiber for the distributed Brillouin sensing application, but only characterized two of the cores for the application. They found that the thermal and strain coefficients differed by < 10% in the first and second cores, but with one parameter being larger and the other being smaller in the first core. They suggested that the change in coefficients largely can be attributed to practical differences in the core materials. Zhao et al. [107] showed that the Brillouin scattering response of multi-core fibers is highly sensitive to bending, thus it makes potential for three-dimensional shape sensors.

**Fig. 10:**
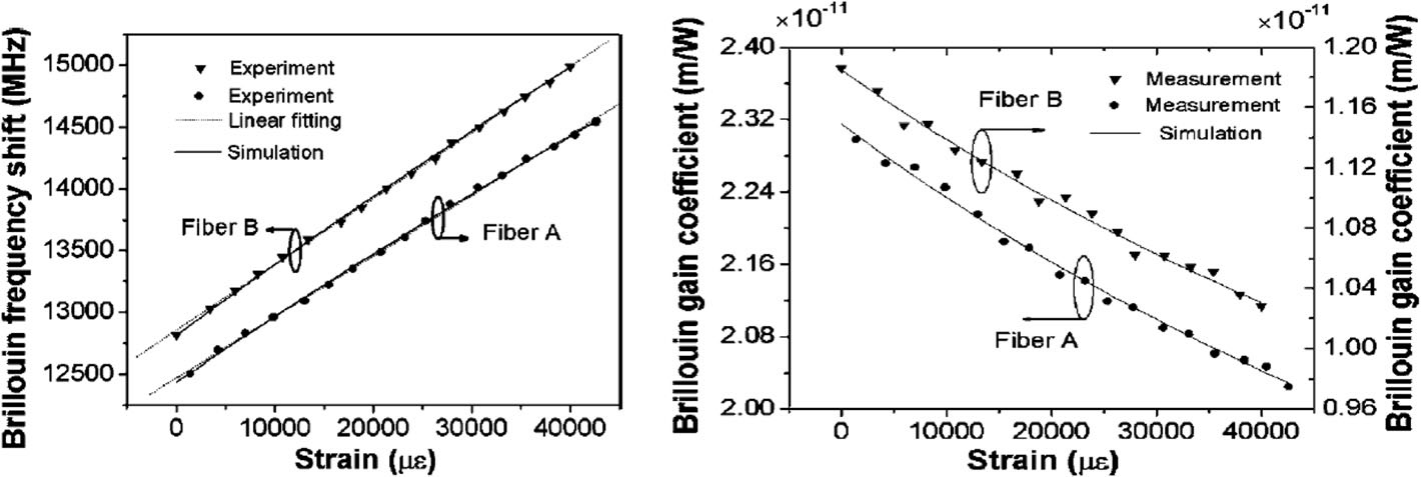
left: BFS for the strain range of *1–40,000*με; right: Brillouin gain coefficient decreases with the strain

Except for SiO_2_ based fiber, distributed Brillouin sensor has been demonstrated in chalcogenide and PMMA fiber for simultaneous temperature and strain sensing [108] based on BOTDA, the PMMA brought large strain range and is compatible to the biomedical applications. The large strain range is important for civil structural monitoring with steel materials. It has been reported 4.3% extension measured by Brillouin gain spectrum using carbon/polyimide coated SMF, [109] as illustrated in Fig. 10. The large strain range of 1–40,000 με showed linear relation with the two types of the specialty fibers with smaller core diameters of 6.3 μm (SMF A, maximum extension of 4.4%), and 8.4 μm (SMF B, maximum extension of 4.2%). Normally with large strain in SMF, Brillouin gain coefficient would drop significantly, however, this is not the case for carbon/polyimide coated SMF, fig d illustrated the Brillouin gain coefficient versus the strain for tape A and B fiber. The gain coefficient is decreased linearly from 2.4 × 10^−11^ *to* 2.1 × 10^−11^ *mW*/*m* over 40,000με, which is about 12.5% reduction over the large strain range. This makes them as ideal candidates for steel structural application, as steel can be extended by 4–6%.

The soft glass As_2_Se_3_ based tapered optical fiber has low Young’s modular comparing with glass fiber and hence it enabled transverse load sensing, namely force sensing via BOTDA system. It is found that the Brillouin frequency shifts of 0.08 ± 0.02 MHz/N are demonstrated over a 2 μm core of As_2_Se_3_ fiber with 100 μm cladding. The mechanism for this force sensor is the high birefringence induced by large difference in the refractive index and Young’s modulus of the core and cladding [110].

## Applications

Infrastructure in North America is aging, the decay process is a big problem faced by many developed countries in the world. After years of service, many structures, such as bridges, tunnels, dams and power plants, are showing severe deterioration, and some of them are being exposed to extreme weather conditions. Over time, these factors can lead to structural distress decreasing the capacity. To ensure safety of these structures and to perform proper maintenance/rehabilitation, there is a need to monitor the structural condition or “health” over time. Also, for important new structures, sensors are often installed during the construction phase so minor degradations can be identified at an early stage.

Structural Health Monitoring (SHM) has been used to identify early signs of potential problems of civil structures to prevent disasters, and conduct needed repairs at the appropriate time to avoid unnecessary costs and reduce economic burden [111]. Thus it is important to have accurate and real time monitoring on the safety assessment of civil structures. The key is to prevent the potential disasters. Currently, such evaluations are carried out by engineers trained in visual inspection, which sometimes can be inaccurate due to the personal experience differences on the safety condition assessment generated by this practice. To increase the inspection efficiency and accuracy, various sensors have been developed and being demonstrated in the field. Among many sensors being used for civil structural monitoring, distributed Brillouin sensors are one of the most promising candidates due to their features of durability, stability, small sizes and insensitivity to external perturbations, which makes them ideal for the long-term health assessment of civil structures.

For SHM the cracks and deformation locations are often unknown in advance, and thus distributed Brillouin sensor can replace many point sensors to find “potential” crack, deformation or buckling locations. This information allows end users to correlate the strains at the different locations to the status of the structures, which enables owners of structures to assess the safety of the structures for various applications, such as (1) oil and gas industry, (2) power industry for power transmission; (3) construction industry; (4) transportation industry, (5) security monitoring. As distributed fiber optic sensors become more cost-effective and the technology advances, the energy, medical, and industrial markets will continuously expand.

## Conclusions

With the high spatial resolution, long distance measurement range and high Brillouin frequency shift measurement accuracy, the Brillouin scattering based distributed fiber sensors can be applied to a large number of applications, such as strain monitoring in pipeline, dames, highway bridges, railway. The Brillouin frequency measurement associated strain and temperature ensure the absolute measurement, which is important for high precision measurement in aerospace and defence. Another feature of the optical fiber is its dielectric nature which makes them immune to the electromagnetic interference, which makes them accessible in harsh environments, such as in airplane and electric power generator. The temperature and strain cross talk challenge can be mitigated by the specialty fibers where multiple Brillouin speaks exist at different acoustic modes, which allows simultaneous temperature and strain sensing. The main challenges for the widespread implementation of the Brillouin based sensors comes from the high price of the interrogation systems. Although the standard optical fiber has a low price of a few cents per meter, the cost of key component of the system is broadband DAQ, which can be mitegated by using low bandwidth DAQ by introducing computational distributed sensing via coding pulse and decorrelation of the receiving traces at different location, as long as coding pulse sequence is know in advance, similar idea is used in Ghost imaging. In this way the bandwidth requirement can be reduced by a factor of ten or higher at the cost of the measurement time, which varied from seconds to a few minutes. However, the overall cost of the sensor system can be dropped by fractional cost.

From the birth of the distributed Brillouin sensor it has been thirty years, many impossible measurement has been demonstrated, the last mile is to bring the lab performance to real world (practical) application that benefit the society and people.

## Data Availability

Data sharing is not applicable to this article as no datasets were generated or analyzed during the current study.
